# Predominance of the c.648G > T *G6PC* gene mutation and late complications in Korean patients with glycogen storage disease type Ia

**DOI:** 10.1186/s13023-020-1321-0

**Published:** 2020-02-11

**Authors:** Yoo-Mi Kim, Jin-Ho Choi, Beom-Hee Lee, Gu-Hwan Kim, Kyung-Mo Kim, Han-Wook Yoo

**Affiliations:** 1 Department of Pediatrics, Chungnam National University Hospital, Chungnam National University, College of Medicine, Daejeon, Korea; 2 Department of Pediatrics, Asan Medical Center Children’s Hospital, University of Ulsan, College of Medicine, Seoul, Korea; 3 Department of Medical Genetics, Asan Medical Center Children’s Hospital, University of Ulsan, College of Medicine, Seoul, Korea

**Keywords:** Glycogen storage disease, *G6PC*, Adult, Complication

## Abstract

**Background:**

Glycogen storage disease (GSD) Ia, caused by mutations in the glucose-6-phosphatase (*G6PC*) gene, is characterized by hepatomegaly, hypoglycemia, lactic acidosis, dyslipidemia, and hyperuricemia. This study aimed to investigate clinical and molecular features and late complications in Korean patients with GSD Ia.

**Results:**

Fifty-four Korean patients (33 males and 21 females) from 47 unrelated families, who were diagnosed with GSD Ia, based on genetic and biochemical data, between 1999 and 2017, were included in this study. The median age at diagnosis was 3.9 years (range: 5 months to 42 years), and the follow-up period was 8.0 ± 6.8 years. Most patients presented with hepatomegaly during infancy, but hypoglycemic symptoms were not predominant. Genetic analysis showed that all the patients had at least one c.648G > T allele. Homozygous c.648G > T mutations in the *G6PC* gene were identified in 34 families (72.3%), and compound heterozygotes with c.648G > T were found in the other families. The allele frequency of c.648G > T was 86.2% (81/94), and p.F51S, p.R83H, p.G122D, p.Y128*, p.G222R, and p.T255A were identified. Of 26 adult patients, 14 had multiple hepatic adenomas, and two were diagnosed with hepatocellular carcinoma. Thirteen patients showed renal complications, and seven patients presented gout, despite preventive allopurinol treatment. Twelve patients had osteoporosis, and two patients had pulmonary hypertension. The final heights were 157.9 cm (standard deviation score: − 3.1) in males and 157.8 cm (standard deviation score: − 0.6) in females.

**Conclusion:**

In our Korean patients with GSD Ia, the most common mutation in the *G6PC* gene was c.648G > T, suggesting a founder effect. Because of only mild hypoglycemia, the patients tended to be diagnosed late. Thus, adult patients with GSD Ia eventually developed diverse and serious complications, which indicates a need for careful monitoring and proper management of this disease.

## Background

Glycogen storage disease (GSD) type Ia (OMIM #232200) is a rare inborn metabolic disorder, caused by glucose-6-phosphatase (G6PC) deficiency, and the overall incidence is considered to be one in 100,000 [1, 2]. This disease leads to defects in glycogenolysis and gluconeogenesis, resulting in the inhibition of glucose production and accumulation of glycogen and fat in the liver, kidney, and intestinal mucosa [2, 3]. The clinical manifestations include hepatomegaly, hypoglycemia, lactic acidosis, hypertriglyceridemia, and hyperuricemia, which are usually manifested in the infantile period. In addition, hepatocellular adenoma and renal dysfunction are frequent late complications [1–3]. Delayed diagnosis and inappropriate therapy lead to many complications, such as growth failure, osteoporosis, refractory gout, renal failure, hepatocellular carcinoma (HCC), and pulmonary hypertension [4–6]. The *G6PC* gene, encoding G6PC, has been mapped to chromosome 17q21 [7], and 110 mutations in *G6PC* have been reported until now. Among them, 70 missense mutations, 14 nonsense mutations, 21 insertions/deletions, and five splicing mutations have been reported (Human Gene Mutation Database: http://www.hgmd.cf.ac.uk). c.648G > T is considered a common mutation in Korean and Japanese patients with GSD Ia [8, 9]; however, there have only been a few reports on clinical characteristics and long-term outcomes of c.648G > T-carrying patients with GSD Ia in a large cohort.

Therefore, this study aimed to investigate clinical and molecular features and late complications in Korean patients with GSD Ia, with a particular focus on c.648G > T-carrying patients.

## Results

### Clinical characteristics and biochemical data

The mean and median age at diagnosis were 9.1 ± 10.7 and 3.9 years (range: 5 months to 42 years), respectively, and the follow-up period was 8.0 ± 6.8 years (Table 1 and Additional file 1: Table S1). Most patients presented with hepatomegaly during infancy and early childhood, whereas nine patients (16.7%) first showed symptoms after 20 years of age. Among late-diagnosed patients, four patients presented with gout, and one patient had dyspnea due to pulmonary hypertension. Three patients visited the hospital for a hepatic mass or hepatomegaly, and one patient had a long-bone fracture and osteoporosis (Table 2). The mean serum glucose level at diagnosis was 79.4 ± 27.7 mg/dL. The serum lactic acid and uric acid levels at diagnosis were 26.1 ± 31.9 mmol/L and 8.0 ± 2.7 mg/dL, respectively. The serum cholesterol (230.4 ± 86.2 mg/dL) and triglyceride (649.7 ± 467.1 mg/dL) levels were also high (Table 1).

**Table 1:** Demographic and clinical characteristics and genotypes of 54 patients with GSD Ia

Characteristic	Total patients (*n* = 54)
Males/Females	33:21
Age at diagnosis	9.1 ± 10.7 (5 months to 42 years)
Age at last visit	16.8 ± 13.1 (13 months to 43 years)
Familial cases	7/47 families (14.9%)
Laboratory findings and height at diagnosis	
Random glucose (mg/dL, RR: 70–120)	79.4 ± 27.7
Lactate (mmol/L, RR: 0.8–2.1)	26.1 ± 31.9
Uric acid (mg/dL, RR: 3.2–7.4)	8.0 ± 2.7
Hemoglobin (mg/dL, RR: 13–16)	10.8 ± 2.0
Cholesterol (mg/dL, RR: 125–220)	230.4 ± 86.2
Triglycerides (mg/dL, RR: 45–150)	649.7 ± 467.1
Height (standard deviation score)	−2.26 ± 2.03
Genotype	
c.648G > T + c.648G > T	34/47 families (72.3%)
c.648G > T + p.G122D	4/47 families (8.5%)
c.648G > T + p.G222R	3/47 families (6.4%)
c.648G > T + p.Y128*	2/47 families (4.3%)
c.648G > T + p.S326P	1/47 families (2.1%)
c.648G > T + p.T255A	1/47 families (2.1%)
c.648G > T + p.F51S	1/47 families (2.1%)
c.648G > T + p.R83H	1/47 families (2.1%)

*RR* reference range

**Table 2:** Long-term complications in 26 adult patients with glycogen storage disease type Ia

Subject	Sex	Recent age (years)	Age at diagnosis (years)	Presenting symptoms	Allele 1	Allele 2	DL	HA	OP	RC	SS (SDS)	AN	DP	GT	PH	HCC
1^a^	F	47.8	29.2	FS, OP	c.648G > T	c.648G > T	+	–	+	–	−1.0	–	–	+	–	–
2	M	43	42	HM, GT	c.648G > T	c.648G > T	+	+	–	+	−2.9	+	–	+	–	+
3	M	43	23	Hp, GT	c.648G > T	c.648G > T	+	–	–	+	−2.7	+	–	+	–	–
4^a^	M	41 (Ex)	31	Hp, Ds	c.648G > T	c.648G > T	+	+	–	–	− 3.3	–	–	+	+	–
5^b^	F	40	36	GT, FS	c.648G > T	c.648G > T	+	–	–	–	0.8	–	+	+	–	–
6^b^	F	37	33	GT	c.648G > T	c.648G > T	+	+	+	+	−3.4	+	+	+	–	–
7	M	36	26	Hp	c.648G > T	c.648G > T	+	+	+	+	−3.7	+	+	+	–	–
8^c^	M	34.1	13.7	Hp, GR	c.648G > T	c.648G > T	+	–	–	–	−1.7	+	–	–	–	–
9	M	28.7	14.8	Hp, GR	c.648G > T	c.648G > T	+	+	+	+	−5.0	+	–	–	–	–
10^d^	F	26.2	8.9	Hp, GR	c.648G > T	c.648G > T	+	+	+	–	−2.2	–	–	–	–	–
11	F	24.1	4.3	Hp, GR	c.648G > T	c.648G > T	+	+	+	+	−1.2	+	+	–	–	–
12^d^	M	21.8	5.8	Hp, GR	c.648G > T	c.648G > T	+	–	+	+	−2.8	–	–	–	–	–
13	M	21.4	20.8	Hp, GR	c.648G > T	c.648G > T	+	+	+	+	−7.5	–	+	–	–	–
14^c^	F	20.8	1.8	FS	c.648G > T	c.648G > T	+	+	+	–	0.4	+	+	–	–	–
15	F	19.1	7	Hp	c.648G > T	c.648G > T	+	–	–	–	1.3	–	+	–	–	–
16	M	18.7	6.1	Hp	c.648G > T	c.648G > T	+	–	–	+	−2.3	+	+	–	–	–
17	F	18.4	2	Hp	c.648G > T	c.648G > T	+	–	–	–	0.4	–	–	–	–	–
18	M	18	13	Hp	c.648G > T	c.648G > T	+	–	–	–	−5.1	–	–	–	–	–
19	M	17.1	3	Hp	c.648G > T	c.648G > T	+	+	–	+	−3.9	–	–	–	–	–
20	M	33.3	8	Hp, HM	c.648G > T	p.G122D	+	+	+	–	−1.8	+	+	+	–	–
21^e^	F	27.5	20.3	Hp, HM	c.648G > T	p.G122D	+	+	–	+	0.4	–	–	–	–	+
22^e^	M	22.8	16	FS	c.648G > T	p.G122D	+	–	+	–	−0.4	–	–	–	–	–
23	M	24.1	2.1	Hp	c.648G > T	p.G222R	+	–	–	–	−2.2	+	+	–	–	–
24	M	19.5	2.5	Hp	c.648G > T	p.G222R	+	+	–	+	−0.9	–	–	–	–	–
25	F	30	17	Hp	c.648G > T	p.S326P	+	+	+	+	−1.7	+	+	–	+	–
26	F	17	4.3	Hp	c.648G > T	p.F51S	+	–	–	–	−0.8	–	–	–	–	–

^a–e^Siblings are indicated by identical superscript letters

*DL* dyslipidemia, *HA* hepatic adenoma, *OP* osteoporosis, *RC* renal complication, *SS* short stature, *SDS* standard deviation score, *AN* anemia, *DP* delayed puberty, *GT* gout, *PH* pulmonary hypertension, *HCC* hepatocellular carcinoma, *Hp* hepatomegaly, *GR* growth retardation, *FS* familial screening, *Ds* dyspnea, *HM* hepatic mass, *Ex* expired

The height standard deviation score (SDS) at diagnosis was − 2.3 ± 2.0 (females: − 1.5 ± 1.9; males: − 2.8 ± 2.1). The midparental height SDS was − 0.2 ± 0.7 (females: − 0.4 ± 0.5; males: − 0.01 ± 0.8). We divided the two groups into early diagnosed patients (age at diagnosis < 3 years) and delayed diagnosed patients (age at diagnosis ≥3 years). At the time of diagnosis, early diagnosed patients were significantly taller than delayed diagnosed patients (− 1.2 ± 1.8 SDS vs. − 3.0 ± 2.0 SDS, *p* < 0.05) and had lower lactic acid levels (8.3 ± 7.6 mmol/L vs. 29.2 ± 34.1 mmol/L, *p* < 0.05). Uncooked cornstarch and allopurinol were prescribed for all patients. The mean dose of uncooked corn starch was 7.2 ± 2.4 g/kg/day. Twenty patients required fibrates or 3-hydroxy-3-methyl-glutaryl-CoA reductase inhibitors for persistent dyslipidemia, despite diet therapy. Nine patients were receiving supplementation of iron because of iron-deficient anemia. Four patients started an angiotensin-converting enzyme inhibitor or an angiotensin receptor blocker for microalbuminuria and hypertension. Bisphosphonate was needed in three adult patients showing severe osteoporosis. After diet control, the biochemical analysis showed that serum glucose (86 ± 20.4 mg/dL), lactic acid (8.2 ± 13.5 mmol/L), and uric acid (6.3 ± 1.8 mg/dL) levels had improved significantly (*p* < 0.05); however, when microalbuminuria and hepatic adenoma occurred in patients, improvements in these complications were not observed. When we compared the height SDS at the latest evaluation between the early diagnosed group and delayed diagnosed group, the early diagnosed patients were taller than the delayed diagnosed patients (− 1.6 ± 0.9 SDS vs. − 2.3 ± 1.9 SDS, *p* < 0.05).

### Molecular analysis

**Fig. 1:**
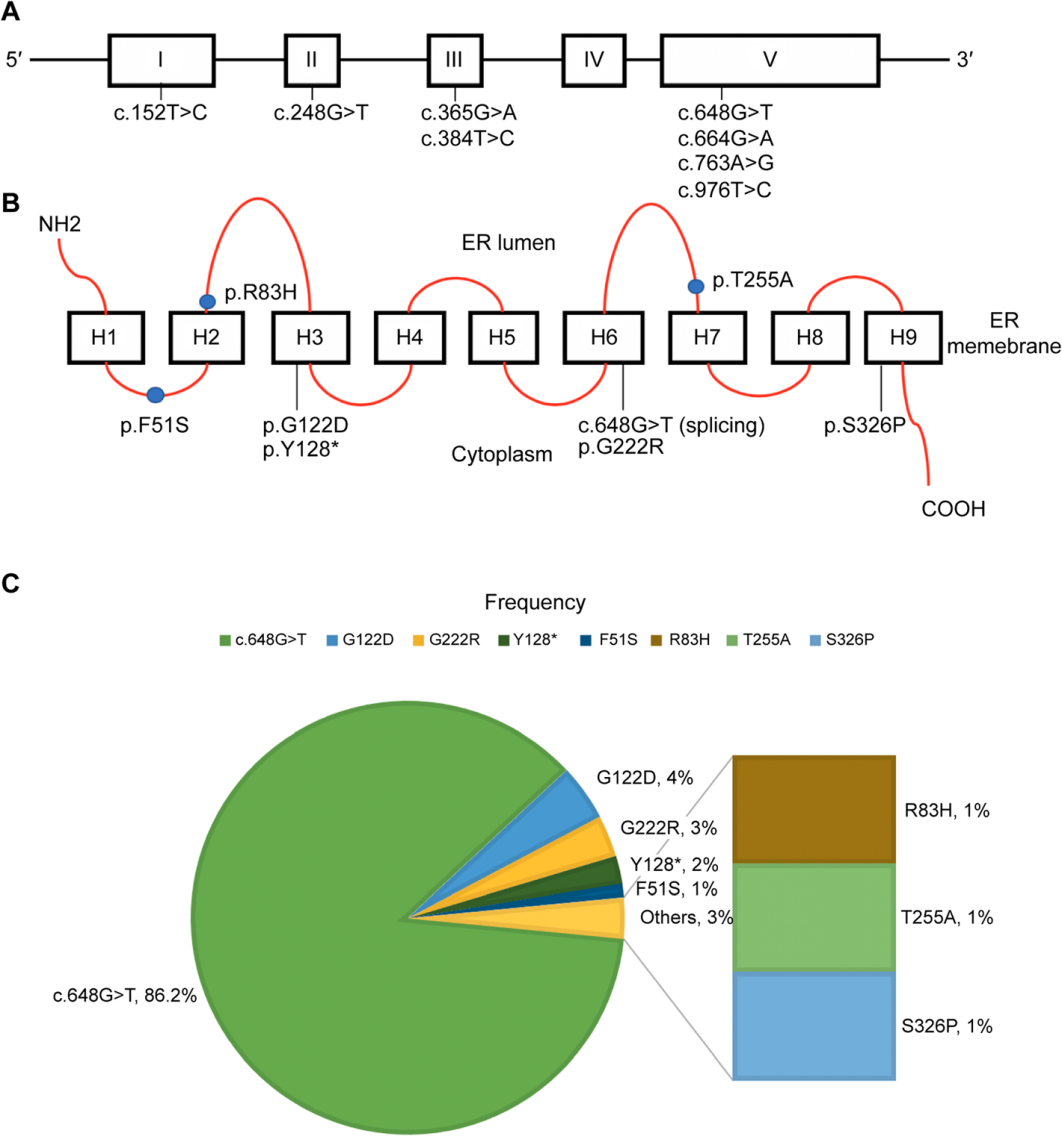
Distribution and frequencies of mutations in the exons and functional domains of G6PC. **a** Eight mutations were identified in exons 1 through 5 of the *G6PC* gene, affecting the function of the encoded enzyme (**b**). **c** Frequencies of various *G6PC* mutations in Korean patients with GSD Ia, showing the predominance of the c.648G > T mutation

Mutation analysis of the *G6PC* gene was conducted in all patients. Seven patients were identified by familial screening. The c.648G > T mutation was most frequently identified, in 81 out of 94 alleles (86.2%; Table 1 and Fig. 1). p.G122D, p.G222R, and p.Y128* were detected in four (4.3%), three (3.2%), and two (2.1%) alleles, respectively. Each of p.F51S, p.R83H, p.T255A, and p.S326P was identified in one allele (1.0%). All eight mutations spanned all exons, except exon 4, and five mutations (62.5%) were identified in exon 5 (Fig. 1). All patients had c.648G > T in at least one allele, and homozygous forms were prevalent in our cohort (72.3% or 34/47 unrelated families). A total of 39 patients were homozygous, and 15 patients were heterozygous for c.648G > T. Comparison between the two groups showed that the homozygous patients were diagnosed later (9.9 ± 11.8 years) than were the patients heterozygous for c.648G > T (6.7 ± 6.9 years; *p* < 0.05). There were no significant differences in biochemical (hemoglobin, lactic acid, triglycerides, glucose, and uric acid) and auxological (height, weight, and body mass index SDSs) findings between the two groups.

### Late complications in GSD Ia

Among 26 adult patients, 15 males and 11 females, 14 patients (54%) were diagnosed at an age of more than 13 years old (Table 2). The frequencies of late complications are summarized in Table 3. Fourteen patients had hepatic adenomas, and the onset age was 19.2 ± 4.4 years (range: 13–27 years). Among them, two patients (14.3%, 2/14 patients with hepatic adenomas) were diagnosed with HCC. Hepatic adenomas were first detected in these two patients at the age of 20 years (subject 2) and 27 years (subject 21). The period between hepatic adenoma and HCC diagnoses was 2 years in subject 2 and 17 years in subject 21. Subject 2, who presented with a large, 13-cm hepatic mass, received right lobectomy, which revealed hepatic cell carcinoma, while subject 21 was lost to follow-up after the HCC diagnosis. Their blood α-fetoprotein (αFP) and chorionic embryonic antigen (CEA) levels were not elevated. The pathologic findings in subject 2 showed Edmondson–Steiner grade 1 and significant disruption of the reticulin framework, supporting the diagnosis of well-differentiated HCC.

**Table 3:** Frequencies of late complications in adult Korean patients with GSD Ia

Total patients (*n* = 26)^a^	n (%)
Hepatomegaly	26 (100%)
Dyslipidemia	26 (100%)
Hepatic adenoma	14 (54%)
Renal complication	13 (50%)
Osteopenia or fracture	12 (46%)
Short stature (SDS: < −2.0)	12 (46%)
Anemia	12 (46%)
Delayed puberty	11 (42%)
Gout	7 (27%)
Pulmonary hypertension	2 (8%)
Hepatocellular carcinoma	2 (8%)

^a^15 males and 11 females

SDS, standard deviation score

Thirteen patients showed renal complications, and the onset age was 23.8 ± 8.5 years (range: 14–37 years). The renal manifestations were microalbuminuria or proteinuria (46.2%; 6/13 patients), micro- or gross hematuria (3/13; 23%), a renal cyst (2/13; 15.4%), medullary calcinosis or ureter stones (3/13; 23%), and renal insufficiency (3/13; 23%). Subject 6, homozygous for c.648G > T, was diagnosed at 34 years of age and presented with gout and stage 4 chronic renal disease at diagnosis (glomerular filtration rate: 18 mL/min/1.73 m^2^). She finally needed hemodialysis, owing to severe hyperkalemia 6 months after the GSD Ia diagnosis. Although this subject had multiple hepatic adenomas, gout, a small height (SDS: − 3.4), and osteoporosis, her older sister (subject 5), who was diagnosed by familial screening, had a normal height (SDS: 0.8), normal renal function, and a mild fatty liver, without hepatic adenomas (Table 2).

Eleven patients (six males and five females) experienced delayed puberty, with the mean age at menarche of 16.9 ± 2.0 years in the females. Seven patients (27%) showed progressive gout, despite allopurinol treatment and diet therapy. Twelve patients (46%) had osteopenia or osteoporosis, as determined by bone densitometry (mean Z-score: − 2.7 ± 0.8), and one patient (subject 1) experienced long-bone fractures twice.

Two patients (subjects 4 and 25) were diagnosed with pulmonary hypertension at the ages of 27 and 22 years, respectively. Subject 4 was treated with beraprost sodium, but he expired at the age of 41 years. Subject 25 had renal failure and was also treated with sildenafil citrate for pulmonary hypertension. Her initial echocardiography showed a pulmonary artery pressure of 81 mmHg and a D-shaped ventricle; the follow-up echocardiography after sildenafil administration demonstrated a mild decrease in the pulmonary artery pressure, to 64 mmHg.

Growth retardation was prominent in the adult male patients; the male patients who had reached adult height had a significantly lower height SDS (SDS: − 3.9 ± 1.8) at diagnosis than female patients (SDS: − 1.7 ± 1.8). Their final height was 157.9 ± 8.9 cm (SDS: − 3.1 ± 1.8) in the males and 157.8 ± 6.8 cm (SDS: − 0.6 ± 1.4) in the females. This might be due to the more delayed diagnosis in males (16.4 ± 10.9 years) compared to females (15.0 ± 13.1 years), although this did not reach statistical significance.

## Discussion

A splice mutation in exon 5 (c.648G > T) of the *G6PC* gene was first reported in 1995, based on the cDNA sequence from the liver of a Japanese patient with GSD Ia, and the activity of this splicing mutant was 18% of the control [10]. Our study demonstrated that c.648G > T was the most common mutation (81/94 alleles; 86.2%) in Korean patients with GSD Ia, which was similar to its frequency in a Japanese patient cohort (88/102 alleles; 86.4%) and different from that in a Chinese population (36%) [4, 11, 12]. A previous report on 13 Korean GSD Ia patients also showed that all individuals carried a c.648G > T mutation as a homozygous or compound heterozygous state except one patient who was a compound heterozygote for the p.G122D and p.Y128* mutations [9]. All adult patients carrying c.648G > T in our study showed a high prevalence of hepatic adenomas. The prevalence of *G6PC* mutations is different depending on the ethnicity. Thus, p.R83H is prevalent in Chinese patients, p.R83C is prevalent in Jewish and eastern European patients, p.Q347* is prevalent in western Europeans, and c.459insTA is prevalent in Mexican and Central American patients [4, 13–15]. Patients carrying c.648G > T, which is prevalent in Japanese and Korean patients, tend to show mild hypoglycemia but are at a high risk for hepatic carcinoma, suggesting that mild hypoglycemia may lead to later diagnosis, poor compliance with diet therapy, and poor metabolic control in GSD Ia [15, 16]. In this study, the homozygous patients were diagnosed later than the heterozygous patients, despite no significant differences in glucose levels (mainly postprandial) at diagnosis. Apart from glucose levels, their biochemical profiles showed elevated serum uric acid, lactic acid, and triglyceride levels at the time of diagnosis. These factors may increase the risk of late complications of GSD in patients with c.648G > T. Nine patients were diagnosed at an age of older than 20 years, of which eight patients were homozygous for c.648G > T. These patients did not experience any severe symptoms of hypoglycemia during infancy and childhood so that severe late complications were their initial manifestations, including a hepatic mass, gout, osteoporosis, and pulmonary hypertension. Since growth retardation was not prominent in patients diagnosed at less than 3 years of age, this also led to late diagnosis in this cohort.

A recent study of Japanese GSD Ia patients with the c.648G > T mutation from 1999 to 2009 showed late diagnosis in male patients who presented with symptoms at the ages of 11 and 9 years, respectively, and one of the 14 patients with hepatic adenoma eventually developed HCC [8]. In addition, there have been more publications about adult GSD Ia patients diagnosed at over 30 years of age [17–19]. They were of diverse heights (-3.2~0.3 SDS) and presented with late complications including liver mass, HCC, or renal failure rather than hypoglycemia.

There are well-established guidelines for regular surveillance of late complications during childhood to adolescence, but it is also important to perform baseline screening for these complications in all patients diagnosed during adulthood [20, 21]. Late complications of GSD1a patients can be reduced by maintaining normoglycemia, which can stabilize metabolism and reduce the synthesis of glucose 6-phosphate, as well as the catabolic status [22]. Diet control improved the biochemical findings in our GSD Ia patients, but it had limited effect on late complications. The height SDS at the latest evaluation can be a predictor of therapeutic effect and compliance with therapy. This data suggested that both adherence to diet control and early diagnosis are critical for adult height outcome.

Chronic lactic acidosis, hyperuricemia, and dyslipidemia are well-known contributing factors to renal insufficiency in GSD Ia, and therefore, patients showing poor compliance with the diet should be closely monitored for microalbuminuria through regular check-ups [23, 24]. Early detection of microalbuminuria and treatment with angiotensin-converting enzyme inhibitors can delay renal deterioration.

Most hepatic adenomas are benign masses, and their size can be reduced through appropriate dietary interventions [20, 21]. However, approximately 10% of hepatic adenomas are known to progress to HCC, and tumor markers, including αFP and CEA, can be negative in these patients with HCC [21, 25]. Therefore, regular radiologic follow-ups in patients with hepatic adenomas are considered the main tool for early detection of HCC [26]. The pathogenesis of HCC in GSD I is not well known. Chronic hypoglycemia-induced hormonal stimulation and accumulation of metabolites in hepatocytes may be underlying factors for malignant transformation of hepatic cells [27]. Recently, an association of a *CTNNB1* mutation with transformation of hepatic adenomas to HCC has been reported in patients with GSD I, indicating potential involvement of a modifying gene [18].

Pulmonary hypertension is a rare but fatal complication in GSD Ia, and its mechanism in GSD I has not been elucidated yet. In our cohort, two late-diagnosed patients showed pulmonary hypertension in their 20s. A regular echocardiography should be performed in adult patients with GSD Ia, and sildenafil, a phosphodiesterase-5 inhibitor, seems to be effective in GSD patients with pulmonary hypertension [28].

Considering the poor prognosis of patients who are diagnosed with GSD Ia at an age older than 20 years, a clinician should not only suspect GSD in patients who present with common endocrine and metabolic issues, including dyslipidemia, hyperuricemia, and osteopenia, but also do familial screening. For stabilizing the metabolic status in patients with GSD I, a continuous glucose monitoring system is considered a useful tool to avoid a hypoglycemic event, which occurs at a serum glucose level of less than 70 mg/dL and triggers a counter-regulatory hormone response, leading to lactic acidosis and synthesis of uric acids and free fatty acids in patients with GSD [29].

Since mild hypoglycemia in patients with GSD Ia does not always guarantee a favorable long-term prognosis, an early diagnosis, even in patients with mild symptoms, is needed to prevent severe complications. Recently, newborn screening has been expanded to include lysosomal storage disorders such as Gaucher disease, Pompe disease, and Fabry disease, as well as mucopolysaccharidosis type I and Niemann–Pick types A/B disease, for early diagnosis and treatment [30]. Considering its irreversible progressive complications, potential treatability with proper management, and genetic background in Korea, GSD Ia can be a candidate for newborn screening in the future. Indeed, neonatal genetic screening for the c.648G > T mutation in the *G6PC* gene may help with early diagnosis in Korean and Japanese populations, although genetic heterogeneity can bring concerns about genetic counseling and unnecessary evaluation [31]. Recently, adeno-associated virus vector-treated GSD Ia in mice showed a possibility to prevent the development of hepatocellular adenoma/carcinoma [32, 33], but was not able to abolish the tumor. Interestingly, Cho et al. [33] demonstrated lower glucose 6-phosphatase expression in Hepatic adenoma and HCC compared to that in non-tumor tissue in a G6PC-knockout mouse after gene therapy despite a similar copy number for the vector genome, and they suggested that the downregulation of glucocorticoid signaling in tumor tissue inhibits gene therapy expression and tumor abrogation. Therefore, it may be difficult to treat late complications through gene therapy alone. However, a new therapeutic strategy targeting the upregulation of glucocorticoid signaling might function in combination with gene therapy for patients with hepatic adenomas or HCC.

Although hepatocyte-targeted gene therapy using an adeno-associated virus vector is in a clinical trial for adult patients with GSD Ia (NCT03517085, NCT03970278; http://www.clinicaltrials.gov), there are many concerning issues and hurdles to overcome. Since the c.648G > T mutation generates aberrant splicing at the transcription level, correction of altered splicing will be possible using antisense oligonucleotide therapy. This genotype-based therapy will be beneficial for more than 80% of patients with GSD Ia in Korea and Japan.

This study entails several limitations. First, this was a retrospective observational study, making statistical analysis problematic because of many uncontrolled confounding factors. Second, biochemical analysis of G6PC was not conducted, making it impossible to correlate biochemical data with the genotype.

## Conclusions

In conclusion, our study showed a prevalent mutation, c.648G > T, in the *G6PC* gene in Korean patients with GSD Ia, and adult patients showed diverse and serious complications, despite mild hypoglycemia and improvement in their biochemical test results. Late diagnosis and overlooked mild hypoglycemia may lead to poor outcomes in Korean patients with GSD. Early detection and proper control of the glucose status in patients with GSD Ia are necessary for a favorable long-term prognosis.

## Methods

### Patients and clinical assessment

A total of 54 patients (33 males and 21 females) from 47 unrelated families, who were diagnosed with GSD Ia between 1999 and 2017, were included in this study (Table 1). Electronic charts were reviewed retrospectively for clinical features, biochemical test results, molecular genetic testing, medications, long-term outcomes, and treatments. Serum glucose, uric acid, lactate, cholesterol, triglycerides, aspartate aminotransferase, alanine aminotransaminase, blood gas, and hemoglobin were monitored to assess the metabolic status. The height, weight, head circumference, and body mass index were tracked to assess growth, and height SDSs were calculated based on the Korean standards for height. Delayed puberty was defined as no secondary sexual manifestations appearing until the age of 14 years for males and 13 years for females. Regular abdominal ultrasound was performed to detect hepatic adenomas. Blood αFP and CEA levels and liver MRI or CT were used when hepatic cell carcinoma was suspected. Bone density tests included DEXA scans and measurement of 25-OH vitamin D levels, and osteopenia and osteoporosis were defined as Z-scores < − 2.0 and < − 2.5, respectively. The DEXA scans were not corrected by bone age or stature. Renal complications were assessed based on the glomerular filtration rate, spot urine microalbumin, and/or protein-to-creatinine ratio, and kidney ultrasound was performed regularly. Pulmonary hypertension was assessed by periodic echocardiography and electrocardiogram.

### Molecular analysis

All mutation analyses of the *G6PC* gene were performed through Sanger sequencing at the Asan Medical Center (Seoul, Korea). Before genetic testing, informed consent was obtained from all patients and their parents. Genomic DNA from peripheral leukocytes was used as a template, and all five exons of the *G6PC* gene were amplified by PCR using intronic primers, designed by the authors, and the GoTaq® colorless master mix (Promega, Madison, WI, USA). Sequencing was performed using the BigDye® Terminator v3.1 cycle sequencing kit (Applied Biosystems, Foster City, CA, USA) on an ABI 3130xl genetic analyzer (Applied Biosystems).

This study was approved by the Research Ethics Board at the Asan Medical Center (approval number: S2019–1025-0001).

### Statistical analysis

Statistical analysis was performed using SPSS for Mac OS version 24.0 (SPSS, Inc., Chicago, IL, USA). Continuous variables were analyzed using a two-sample *t*-test or Mann–Whitney *U*-test.

## Supplementary information

**Additional file 1: Table S1.** Genotype and age at diagnosis for 54 Korean patients with glycogen storage disease type Ia.

## Data Availability

Not applicable.

## Abbreviations

CEA: Chorionic embryonic antigen
G6PC: Glucose-6-phosphatase
GSD: Glycogen storage disease
HCC: Hepatocellular carcinoma
SDS: Standard deviation score
αFP: α-Fetoprotein
