# Supplementary material for: Predominance of the c.648G > T G6PC gene mutation and late complications in Korean patients with glycogen storage disease type Ia
Source: Orphanet J Rare Dis. 2020 Feb 11;15:45. doi: 10.1186/s13023-020-1321-0 (PMC7014716; doi:10.1186/s13023-020-1321-0)
Supplement: Supplementary file 1 — Additional file 1: Table S1. Genotype and age at diagnosis for 54 Korean patients with glycogen storage disease type Ia. [file 13023_2020_1321_MOESM1_ESM.docx]

**Table S1.** Genotype and age at diagnosis for 54 Korean patients with glycogen storage disease type Ia.

| **Subject** | **Sex** | **Recent age (years)** | **Age at diagnosis (years)** | **Presenting symptoms** | **Allele 1** | **Allele 2** |
| --- | --- | --- | --- | --- | --- | --- |
| 1^a^ | F | 47.8 | 29.2 | Family screening, osteoporosis | c.648G>T | c.648G>T |
| 2 | M | 43 | 42 | Gout, hepatic mass | c.648G>T | c.648G>T |
| 3 | M | 43 | 23 | Gout, hepatomegaly | c.648G>T | c.648G>T |
| 4^a^ | M | 41 (Ex) | 31 | Hepatomegaly, dyspnea | c.648G>T | c.648G>T |
| 5^b^ | F | 40 | 36 | Gout, family screening | c.648G>T | c.648G>T |
| 6^b^ | F | 37 | 33 | Gout | c.648G>T | c.648G>T |
| 7 | M | 36 | 26 | Hepatomegaly | c.648G>T | c.648G>T |
| 8^c^ | M | 34.1 | 13.7 | Hepatomegaly, growth retardation | c.648G>T | c.648G>T |
| 9 | M | 28.7 | 14.8 | Hepatomegaly, growth retardation | c.648G>T | c.648G>T |
| 10^d^ | M | 21.8 | 5.8 | Hepatomegaly, growth retardation | c.648G>T | c.648G>T |
| 11 | F | 24.1 | 4.3 | Hepatomegaly, growth retardation | c.648G>T | c.648G>T |
| 12^d^ | F | 26.2 | 8.9 | Hepatomegaly, growth retardation | c.648G>T | c.648G>T |
| 13 | M | 21.4 | 20.8 | Hepatomegaly, growth retardation | c.648G>T | c.648G>T |
| 14^c^ | F | 20.8 | 1.8 | Family screening | c.648G>T | c.648G>T |
| 15 | F | 19.1 | 7 | Hepatomegaly | c.648G>T | c.648G>T |
| 16 | M | 18.7 | 6.1 | Hepatomegaly | c.648G>T | c.648G>T |
| 17 | F | 18.4 | 2 | Hepatomegaly | c.648G>T | c.648G>T |
| 18 | M | 18 | 13 | Hepatomegaly | c.648G>T | c.648G>T |
| 19 | M | 17.1 | 3 | Hepatomegaly, growth retardation | c.648G>T | c.648G>T |
| 20 | M | 33.3 | 8 | Hepatomegaly, hepatic mass | c.648G>T | p.G122D |
| 21^e^ | F | 27.5 | 20.3 | Hepatic mass, hepatic adenoma | c.648G>T | p.G122D |
| 22^e^ | M | 22.8 | 16 | Family screening | c.648G>T | p.G122D |
| 23 | M | 24.1 | 2.1 | Hepatomegaly | c.648G>T | p.G222R |
| 24 | M | 19.5 | 2.5 | Hepatomegaly | c.648G>T | p.G222R |
| 25 | F | 30 | 17 | Hepatomegaly | c.648G>T | p.S326P |
| 26 | F | 17 | 4.3 | Hepatomegaly | c.648G>T | p.F51S |
| 27 | M | 16.1 | 1.2 | Hepatomegaly, diarrhea | c.648G>T | c.648G>T |
| 28 | F | 16.1 | 1 | Hepatomegaly | c.648G>T | c.648G>T |
| 29 | F | 16 | 2.2 | Hepatomegaly | c.648G>T | c.648G>T |
| 30 | M | 14.7 | 2.4 | Hepatomegaly | c.648G>T | c.648G>T |
| 31 | M | 8.2 | 1.3 | Lactic acidosis, seizures | c.648G>T | c.648G>T |
| 32^f^ | M | 7.3 | 1.8 | Hepatomegaly, hypoglycemia | c.648G>T | c.648G>T |
| 33 | M | 10 | 5.4 | Epistaxis, hepatomegaly | c.648G>T | c.648G>T |
| 34 | M | 6 | 5 | Hepatomegaly | c.648G>T | c.648G>T |
| 35 | F | 5.5 | 3.8 | Hepatomegaly | c.648G>T | c.648G>T |
| 36^f^ | M | 5 | 0.4 | Family screening | c.648G>T | c.648G>T |
| 37 | M | 4 | 0.9 | Hepatomegaly | c.648G>T | c.648G>T |
| 38 | F | 2 | 1 | Hepatomegaly | c.648G>T | c.648G>T |
| 39 | M | 2 | 2 | Hepatomegaly | c.648G>T | c.648G>T |
| 40 | F | 3.2 | 2.8 | Hepatomegaly | c.648G>T | c.648G>T |
| 41 | F | 1.0 | 0.6 | Hepatomegaly | c.648G>T | c.648G>T |
| 42 | F | 1.0 | 0.5 | Hepatomegaly | c.648G>T | c.648G>T |
| 43 | M | 1.6 | 0.4 | Hepatomegaly | c.648G>T | c.648G>T |
| 44 | F | 1.7 | 1.7 | Hepatomegaly | c.648G>T | c.648G>T |
| 45 | M | 1 | 1 | Hepatomegaly | c.648G>T | c.648G>T |
| 46 | M | NA | 18 | Hepatomegaly | c.648G>T | c.648G>T |
| 47 | F | 16 | 16 | Hepatomegaly | c.648G>T | p.G122D |
| 48^g^ | M | 3 | 3 | Hepatomegaly | c.648G>T | p.G122D |
| 49^g^ | M | 4 | 4 | Hepatomegaly | c.648G>T | p.G122D |
| 50 | M | 7.9 | 0.7 | Hepatomegaly | c.648G>T | p.Y128* |
| 51 | F | 2.4 | 0.7 | Hepatomegaly | c.648G>T | p.Y128* |
| 52 | M | 6.7 | 1.4 | Hepatomegaly | c.648G>T | p.G222R |
| 53 | M | 3.0 | 2.3 | Hepatomegaly | c.648G>T | p.T255A |
| 54 | M | 2.8 | 2.4 | Hepatomegaly, diarrhea | c.648G>T | p.R83H |

^a–g^Sibling cases are indicated by identical superscript letters.

Ex, expired; NA, not available
